# Solar PVT-assisted high-temperature CO_2_ heat pump with thermal storage: Design for net-zero electricity operation and ethical sustainability

**DOI:** 10.1371/journal.pone.0349803

**Published:** 2026-06-02

**Authors:** Yuan Ma, Yaxiong Wang, Yu Ma, Ziyang Cheng, Di Zhong

**Affiliations:** 1 Department of Philosophy, School of Humanities and Social Science, Xi’an Jiaotong University, Xi’an, China; 2 Shanghai Police College, Shanghai, China; 3 School of Mechanical Engineering, Xi’an University of Science and Technology, Xi’an, China; 4 Huaneng Clean Energy Research Institute, Beijing, China; Aalto University, FINLAND

## Abstract

To build a clean, low-carbon and efficient energy utilization system, solar photovoltaic–thermal (PVT) assisted heat pump technologies are promising for supplying high-temperature heat in industrial sectors and accelerating the green transition of the energy system. By targeting net-zero daily electricity use and reduced reliance on fossil fuels, such systems can also support ethically sustainable industrial process heat. In this study, a solar PVT-assisted high-temperature CO_2_ heat pump system with a hot-water storage tank is proposed and analyzed. The PVT field simultaneously generates electricity to drive the compressor and recovers photovoltaic waste heat to charge the thermal storage, which serves as the low-temperature heat source of a transcritical CO_2_ heat pump. This work presents a dynamic co-design of an integrated PVT-storage-heat-pump system to achieve net-zero daily electricity operation, with explicit consideration of ethical sustainability in industrial heat supply. A dynamic mathematical model is developed and applied to a typical meteorological day in Xi’an, China, to provide 10 kW continuous heat above 100 °C over 24 h. The effects of the number of PVT modules, storage tank volume and initial storage temperature on system behavior are investigated. Results show that at least 32 PVT modules are required to guarantee 24 h stable operation, with the storage temperature and heat pump COP (Coefficient of performance) exhibiting a decrease–increase–decrease trend throughout the day; as the initial storage temperature increases from 25 °C to 50 °C, the average COP rises from 3.59 to 4.37. For a given number of PVT modules, the net daily electrical output increases with storage volume and significantly increases with the initial storage temperature, varying from −6.06 kWh to 2.32 kWh within the investigated range, and reaching its maximum at a storage volume of 5 m³ and an initial temperature of 50 °C. A storage volume of 4.4 m³ and an initial temperature of 42 °C yield a net daily electrical output of approximately zero, i.e., the electricity generated by the PVT field fully compensates the compressor consumption. Under this self-sufficient condition, the maximum instantaneous electrical efficiency gain of the PVT modules compared with a standalone PV array is 5.03%, while the maximum hourly PVT electrical output and net electrical output reach 5.91 kWh and 3.43 kWh, respectively.

## 1. Introduction

Energy is a fundamental material basis for human survival and sustainable development. In the “14th Five-Year Plan” of China, the government emphasises promoting an energy revolution and building a clean, low-carbon, safe and efficient energy system [1]. Solar energy, as one of the most important primary energy sources, plays a crucial role in achieving these targets by increasing its share in the primary energy mix.

Currently, solar energy utilization can be broadly categorized into photovoltaic (PV) power generation [2] and solar thermal applications [3]. With the rapid development of PV technologies, the cost of PV electricity has continuously decreased, and the cumulative global installed PV capacity reached approximately 760 GW by 2020 [4]. However, conventional PV modules convert only a fraction of the incident solar irradiance into electricity, while the remainder is dissipated as heat, resulting in elevated module temperatures. Insufficient heat dissipation reduces electrical efficiency and accelerates material aging, thereby shortening the service life of PV modules. Recent studies have also shown that PV output can be further enhanced through optical design, such as flat mirrors and bifacial configurations, although these approaches also make irradiance distribution and system layout more important [5,6].

Solar photovoltaic–thermal (PVT) technology combines PV power generation and solar thermal collection in a single device, where a working fluid extracts waste heat from the PV rear side to control the operating temperature and simultaneously delivers useful heat for thermal applications [7]. The basic concept of PVT collectors has long been established, with early studies showing that PV cooling can simultaneously improve electrical performance and recover useful heat [8]. PVT systems therefore improve the overall energy utilization efficiency, and are particularly attractive when both electricity and heat are required. Bashar Shboul et al. [9] analyzed a photovoltaic thermal collector integrated with a horizontal axis wind turbine for combined heat and power supply. Their results showed that the system could effectively reduce CO_2_ emissions and generate considerable income for users. In addition, novel solar photovoltaic thermal systems have been proposed to enhance PVT performance, such as those employing finned cooling channels [10] or zigzag channel structures [11]. Moreover, studies have confirmed that the materials used in photovoltaic panels significantly influence PVT efficiency. For instance, using porous flax fiber materials dispersed in pure water as a cooling fluid enabled the PVT system to achieve a thermal efficiency of 69.58% [12].

Heat pumps are widely recognized as an energy-efficient technology for upgrading low-grade heat to higher temperature levels. Solar-assisted heat pump systems can be effectively coupled with PVT collectors, where the PVT waste heat is used as the low-temperature source of the heat pump [13–16]. Previous studies on PVT-assisted heat pumps have mainly focused on domestic hot-water applications with relatively low supply temperatures. For example, Chu et al. [14] experimentally investigated a PVT air-source heat pump system for combined heat and power. Under combined operation, the PV cell temperature was reduced by 9.8 °C on average compared with a standalone PV array, and the electrical conversion efficiency increased by 17.53%. Liu et al. [15] performed seasonal simulation of a direct-expansion PVT heat pump water heating system considering different configurations; they reported that reducing the compressor theoretical displacement improved system COP, while extending the required heating time. Yao et al. [16] analyzed the performance of a direct-expansion PVT heat pump, and found that about 21.4% of the electricity generated by the PVT field could be exported to the grid after covering the compressor demand.

Although previous studies have demonstrated the feasibility and benefits of solar PVT collectors and PVT-assisted heat pump systems, several limitations remain. Most experimental and numerical works still focus on domestic hot water or low-temperature space heating (typically below 60–70 °C), while high-temperature industrial process heat above 100 °C has been much less explored. Many investigations also consider the PVT collector and the heat pump separately, or treat the solar input in a simplified manner, without resolving the dynamic coupling among PVT electrical–thermal behaviour, thermal storage and the heat pump cycle over a full day. Furthermore, the explicit impact of thermal storage sizing and initial thermal conditions on net daily electricity use is often neglected: optimization studies usually target COP, seasonal performance factors or economic indicators, rather than design conditions for net-zero daily electricity consumption in PVT-assisted high-temperature systems. Finally, the ethical and sustainability implications of these configurations for decarbonizing industrial heat supply are rarely discussed in a structured way, despite their relevance to energy justice and responsible engineering practice.

In view of the above research gaps, this study develops and evaluates an integrated solar PVT-assisted high-temperature CO_2_ heat pump system with thermal storage. The system is designed to provide continuous high-grade heat. The study also examines its potential to approach net-zero daily electricity use for industrial applications. The main contributions of this work are as follows:

(1) A solar PVT-assisted transcritical CO_2_ heat pump system with hot-water storage is proposed for heat supply above 100 °C. The system is intended to reduce carbon emissions from industrial process heating. It also aims to lower reliance on external electricity.
(2) A dynamic mathematical model is developed for the integrated system. The model describes the electrical and thermal performance of the PVT collectors. It also captures the energy balance of the storage tank. In addition, it predicts the thermodynamic behavior of the transcritical CO_2_ heat pump under time-varying weather conditions.
(3) The system performance is evaluated for a typical meteorological day in Xi’an, China. The analysis considers a continuous 10 kW heat demand above 100 °C. Sensitivity analyses are performed for the number of PVT modules, the storage tank volume, and the initial storage temperature. Their effects on heat pump COP, PVT electrical efficiency, and net daily electrical output are quantified.
(4) The relationships among heat pump COP, PVT electrical efficiency, and net daily electrical output are analyzed. Based on these results, design guidelines are proposed for net-zero daily electricity operation. The implications of the system are also discussed from the perspective of ethical sustainability in industrial energy use.

These findings provide methodological support for modelling and evaluating solar-assisted high-temperature heat pump systems. They also offer practical guidance for system design aimed at low-electricity or net-zero-electricity industrial heat supply.

## 2. System description

### 2.1. Overall system configuration

**Fig 1:**
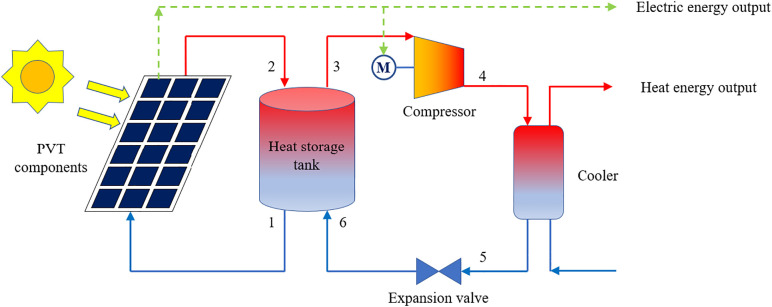
Schematic diagram of the proposed solar PVT-assisted heat pump system with thermal storage, including the PVT collectors, heat storage tank, compressor, and energy flow pathways.

The proposed solar PVT-assisted high-temperature CO_2_ heat pump system with thermal storage is schematically illustrated in Fig 1. The system mainly comprises a PVT field, a hot-water storage tank, a CO_2_ compressor, a gas cooler and an expansion valve. On the electrical side, the PV layer of the PVT modules converts part of the incident solar irradiance into electricity. This electricity is primarily used to drive the CO_2_ compressor; any surplus electricity can be supplied to external loads or the grid. On the thermal side, the PVT modules recover waste heat from the PV cells via a cooling water circuit. The heated water is stored in the tank and serves as the heat source for the transcritical CO_2_ heat pump. The CO_2_ working fluid absorbs heat from the storage tank in the evaporator heat exchanger, is compressed to a supercritical pressure, and then releases high-temperature heat to industrial users through the gas cooler. After throttling in the expansion valve, CO_2_ returns to the evaporator and closes the cycle. The integration of thermal storage decouples the solar collection from the heat pump operation. During periods with sufficient solar irradiance, the PVT field both generates electricity and charges the tank; during low-irradiance or night-time periods, the heat pump continues to operate using the stored thermal energy, thereby maintaining stable heat supply.

### 2.2. PVT module structure

**Fig 2:**
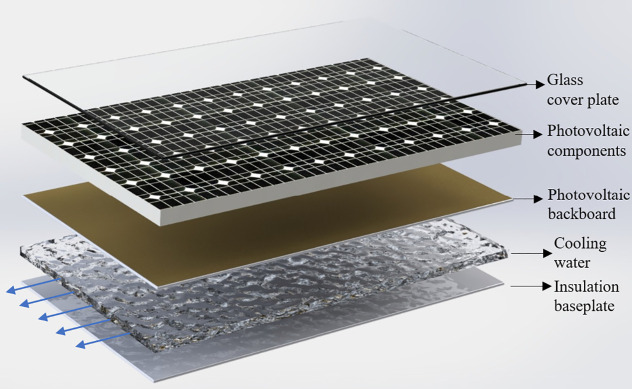
Structure of the PVT components.

The structure of a single PVT module is shown in Fig 2. From top to bottom, the layers include a glass cover, a PV cell layer, a PV back-sheet, a water channel and an insulation plate [17]. The cooling water flows through the channel between the back-sheet and the insulation plate and extracts waste heat from the PV cells.

The main structural parameters of a single PVT module are listed in Table 1 [18]. The aperture area of the PV module is 1.32 m². The mass flow rate of cooling water through each module is 0.016 kg/s. The effective transmittance–absorptance product of the absorber surface is 0.66. The thicknesses of the glass cover, PV layer, back-sheet, water channel and insulation layer are 0.003 m, 0.0003 m, 0.0005 m, 0.05 m and 0.05 m, respectively.

**Table 1:** Main structural parameters of a single PVT component.

Items	Values
Area of PVT component	1.32 m^2^
Mass flow rate of cooling water	0.016 kg/s
Effective transmission rate and absorption rate	0.66
Thickness of the glass cover plate	0.003 m
thickness of the photovoltaic panel	0.0003 m
Thickness of the photovoltaic backboard	0.0005 m
Thickness of the cooling water layer	0.05 m
Thickness of the insulation baseplate	0.05 m

When solar irradiance is strong, the cooling water not only reduces the PV operating temperature, enhancing electrical efficiency, but also collects scattered and reflected radiation from the back-sheet. Compared with a conventional water-source heat pump using ambient water, the PVT-assisted system achieves a higher evaporation temperature and improved thermodynamic performance.

## 3. Mathematical model and performance indicators

To analyze the proposed system, a dynamic mathematical model is developed with the following assumptions:

(1) The system is treated as quasi-steady within each simulation time step, i.e., thermodynamic states are assumed steady over the time increment.
(2) The water in the storage tank is perfectly mixed, so that temperature stratification is neglected and a uniform bulk temperature is used.
(3) The outlet temperature of the storage tank is equal to the inlet temperature of the PVT field, and the thermal inertia of connecting pipes is neglected.
(4) Pressure losses in pipes and heat exchangers are neglected, and only the main pressure levels of the transcritical CO_2_ cycle are considered.
(5) A fixed degree of superheat is maintained at the compressor inlet to avoid liquid ingestion and ensure safe operation.

### 3.1. PVT electrical and thermal model

The electrical efficiency of the PV layer in the PVT module varies with cell temperature and is expressed as [19]:


$$\eta =\eta_{r}\left[\text{1}-\beta \left(T_{c}-T_{r}\right)\right]$$ (1)

where *η* is the instantaneous electrical efficiency; *η*_r_ is the reference efficiency at *T*_r_ = 25 °C (taken as 19.1% in this study); *β* is the temperature coefficient (0.0045 °C ⁻ ¹); and *T*_c_ is the PV cell temperature (°C).

The useful thermal power gained by the cooling water flowing through the PVT module is


$$Q_{u}={\dot{m}}_{w}C_{p,w}\left(T_{\text{2}}-T_{\text{1}}\right)$$ (2)

where ${\dot{m}}_{w}$ is the mass flow rate of water (kg/s); $C_{p,w}$ is the specific heat capacity of water (J/(kg·K)); and *T*_1_ and *T*_2_ are the inlet and outlet temperatures of the cooling water (°C).

The absorbed thermal power can also be estimated by an energy balance similar to that of a flat-plate solar collector [20]:


$$Q_{u}=F_{R}A_{col}[h_{p\text{1}}h_{p\text{2}}{\left(\alpha \tau \right)}_{eff}\;I-U_{L}\left(T_{\text{1}}-T_{amb}\right)]$$ (3)

where $A_{col}$ is the module area (m²); $F_{R}$ is the heat removal factor; ${\left(\alpha \tau \right)}_{eff}$ is the effective transmittance–absorptance product; *I* is the incident solar irradiance (W/ m²); *U*_L_ is the overall heat loss coefficient (W/m²/K); and *T*_amb_ is the ambient air temperature (°C).

The heat removal factor (F_R_) is given by [20]:


$$F_{R}=\frac{{\dot{m}}_{w}C_{p,w}}{A_{col}U_{L}}\left[\text{1}-exp\left(\frac{-A_{col}U_{L}F^{\prime }}{{\dot{m}}_{w}C_{p,w}}\right)\right]$$ (4)

The overall heat loss coefficient is


$$U_{L}=U_{gw}+{\left(\frac{L_{ins}}{K_{ins}}+\frac{\text{1}}{h_{conv}}\right)}^{-\text{1}}$$ (5)

$$h_{conv}=\text{2}.\text{8}+\text{3}\times V_{\text{wind}}$$ (6)

where $U_{gw}$ is the overall heat transfer coefficient from the PV panel to the cooling water ($\text{W}/({\text{m}}^{\text{2}}\cdot \text{K})$); $L_{ins}$ is the thickness of the insulation plate (m); $K_{ins}$ is the thermal conductivity of the insulation plate (W/(m·K)); $h_{conv}$ is the convective heat transfer coefficient at the outer surface of the module ($\text{W}/({\text{m}}^{\text{2}}\cdot \text{K})$); and $V_{\text{wind}}$ is the local wind speed (m/s).

### 3.2 Storage tank model

The energy balance of the perfectly mixed storage tank can be written as:


$$Q_{u}=M_{w}C_{p,w}\frac{dT_{w}}{dt}+{\left(UA\right)}_{Tank}\left(T_{w}-T_{amb}\right)+{\dot{m}}_{{co}_{\text{2}}}\left(h_{\text{3}}-h_{\text{6}}\right)$$ (7)

where *M*_w_ is the total water mass in the tank (kg); *T*_W_ is the uniform storage temperature (°C); U is the heat loss coefficient (W/m²/K), A is the heat exchange area (m^2^), ${\dot{m}}_{{co}_{\text{2}}}\;$is the mass flow rate of CO_2_ in the system (kg/s); and *h*_3_ and *h*_6_ are the specific enthalpies of CO_2_ at the storage tank inlet and outlet (J/kg), respectively.

By combining equations (3) and (7), the tank temperature can be calculated as follows:


$$\frac{dT_{w}}{dt}=\frac{F_{R}A_{col}[h_{p\text{1}}h_{p\text{2}}{\left(\alpha \tau \right)}_{eff}\;I-U_{L}\left(T_{\text{1}}-T_{amb}\right)]-{\left(UA\right)}_{Tank}\left(T_{w}-T_{amb}\right)-{\dot{m}}_{{co}_{\text{2}}}\left(h_{\text{3}}-h_{\text{6}}\right)}{M_{w}C_{p,w}}$$ (8)

### 3.3. Transcritical CO_2_ heat pump model

The CO_2_ heat pump cycle consists of an evaporator using storage hot water as the heat source, a compressor, a gas cooler and an expansion valve. The compressor outlet enthalpy is calculated assuming a given isentropic efficiency:


$$h_{\text{4}}=h_{\text{3}}+\left(h_{\text{4s}}-h_{\text{3}}\right)/\eta_{is}$$ (9)

where *h*_3_ and *h*_4_ are the specific enthalpies at compressor inlet and outlet (J/kg), respectively; *h*_4s_ is the outlet enthalpy for isentropic compression; and *η*_is_ is the compressor isentropic efficiency.

The compressor power input is


$$W_{c}=h_{\text{3}}+\left(h_{\text{4s}}-h_{\text{3}}\right)/\eta_{is}$$ (10)

The expansion valve is modelled as an isenthalpic process:


$$h_{\text{5}}=h_{\text{6}}$$ (11)

The useful heating capacity delivered to the industrial user at the gas cooler is


$$Q_{h}={\dot{m}}_{{co}_{\text{2}}}\left(h_{\text{4}}-h_{\text{5}}\right)$$ (12)

### 3.4. Performance indicators

Three key performance indicators are used to evaluate the PVT-assisted transcritical CO_2_ heat pump system.

1. Coefficient of performance (COP) of the heat pump


$$COP=\frac{Q_{h}}{W_{c}}$$ (13)

2. Electrical generation gain of the PVT field

Because the cooling water lowers the PV cell temperature compared with a standalone PV array, the PVT system yields additional electricity. The relative electrical generation gain is defined as [15]:


$$B=\frac{P_{PVT}-P_{PV}}{P_{PV}}$$ (14)

3. Net electrical output of the system


$$W=\sum_{t}\left(P_{PVT}-W_{c}\right)\times t$$ (15)

### 3.5. Model validation

To ensure the reliability of the proposed model, the main models are validated against published experimental data and standard thermodynamic calculations. For the PVT model, we validated the simulated electrical output against published experimental data for a representative PVT system over a typical day [20]. Using the same irradiance, ambient temperature and operating conditions as reported in the reference, the RMS (root-mean-square) deviation between the simulated and measured daily electrical generation is 5.1%, which is essentially identical to the RMS value of 5.3% reported in the literature for the comparison between their model and experiments. This close agreement indicates that the proposed PVT model can reliably reproduce the electrical performance and daily power output of the PVT field under realistic operating conditions.

**Fig 3:**
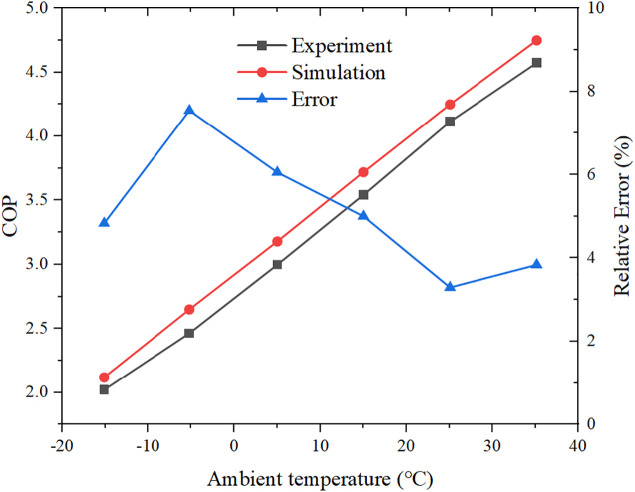
The validation of the transcritical CO2 heat pump cycle.

For the transcritical CO_2_ heat pump, the cycle model was validated against published experimental data for a CO_2_ heat pump water heater [21]. By setting the simulation parameters identical to those in the reference and considering the test case with a hot-water outlet temperature of 55 °C, we compared the simulated COP with the experimental COP over a range of ambient temperatures (Fig 3). The relative error between the simulated and measured COP values remains within 8% for all operating points, which confirms the acceptable accuracy of the constructed CO_2_ heat pump model for system-level analysis.

## 4. Results and discussion

**Fig 4:**
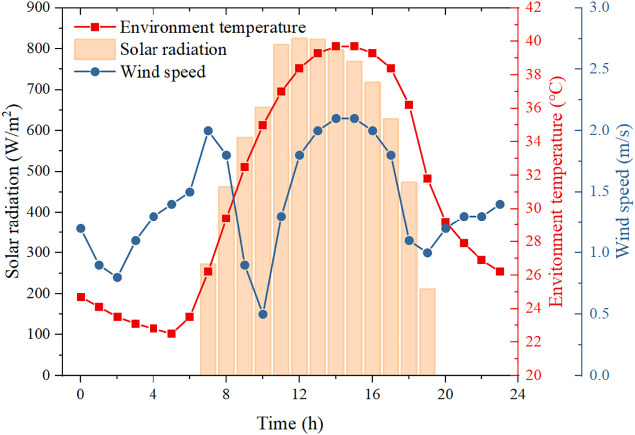
Variations of solar irradiance, ambient temperature, and wind speed during a typical day used as boundary conditions for the system simulation.

The system is designed to provide continuous 10 kW heat at a supply temperature above 100 °C over a 24h typical day. Xi’an City in Shaanxi Province, China, is selected as the case study location. Hourly meteorological data of the typical day, including solar irradiance, ambient temperature and wind speed, are shown in Fig 4. The maximum solar irradiance reaches 825 W/m^2^ and the highest ambient temperature is 39.7 °C.

The dynamic simulation is implemented in MATLAB. At each time step, the CO_2_ thermophysical properties are obtained from REFPROP 10.0 (NIST Reference Fluid Thermodynamic and Transport Properties Database: Version 10). The main design and operating parameters are summarized in Table 2. The system operates from 00:00–23:59 with a design heating capacity of 10 kW. The base-case configuration employs 32 PVT modules and a storage tank volume of 4 m³, with an initial storage temperature of 25 °C.

**Table 2:** The main design and operating parameters of the proposed system.

Items	Values (range)
Power of heat energy supply	10kW
Temperature of heat energy supply	>100°C
Aperture area per PVT module	1.32 m^2^
Number of PVT components	32
Volume of heat storage tank	4m^3^ (3–5 m^3^)
Initial temperature of heat storage tank	25°C (25–50°C)
System operation time	0:00-23:59

**Fig 5:**
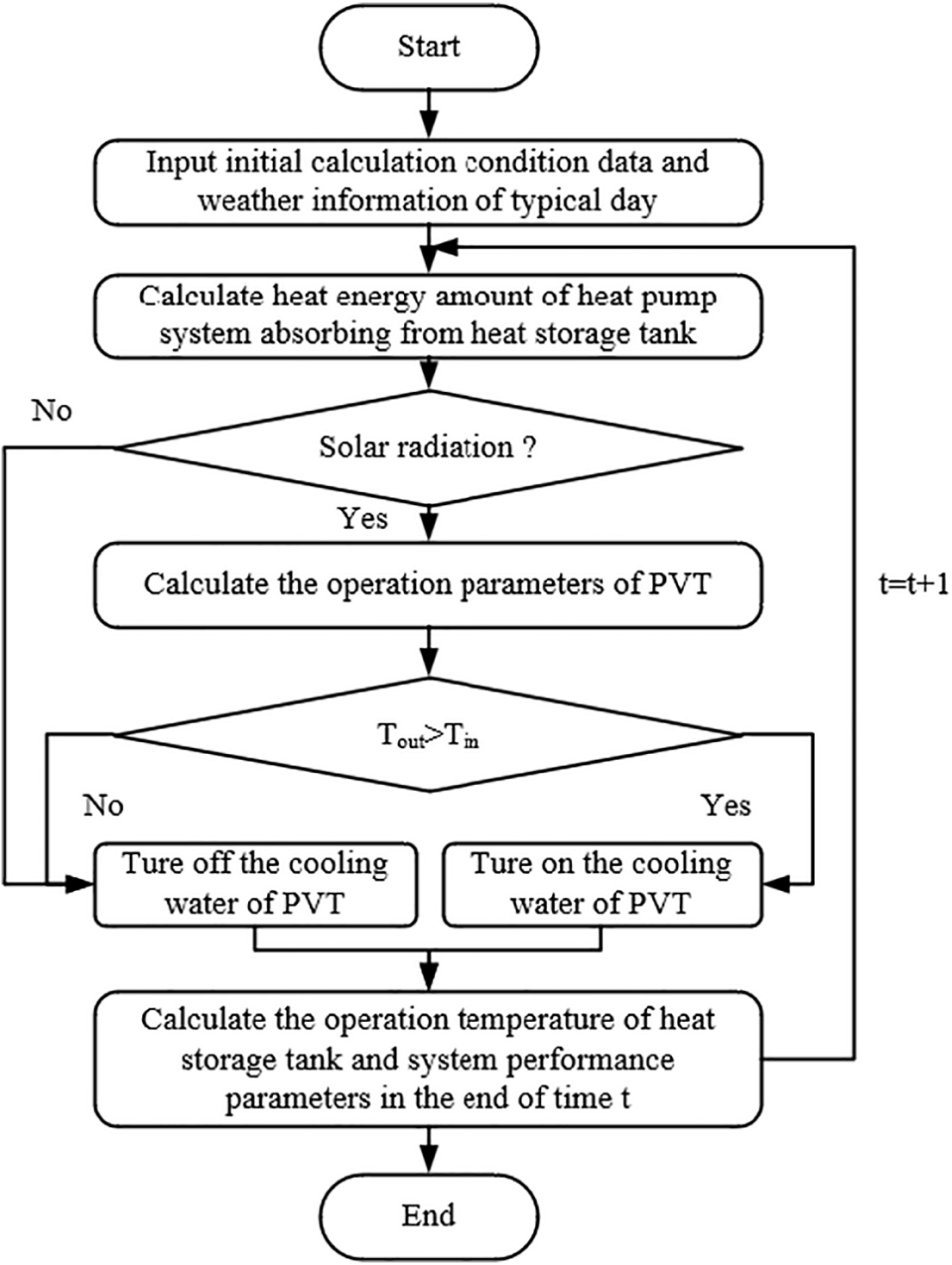
Calculation process in system performance simulation.

The overall computational procedure is illustrated in Fig 5. For each time step, the transcritical CO_2_ cycle is first solved based on the current storage temperature and load conditions. Subsequently, the thermal and electrical performance of the PVT modules is evaluated according to the solar irradiance and inlet water temperature. The storage tank temperature is updated by the energy balance and used as the initial temperature for the next time step. System performance indicators such as COP, PVT electrical efficiency, electrical generation gain and net electrical power are then obtained.

Sensitivity analyses are carried out by varying one parameter at a time (PVT module number, storage tank volume or initial storage temperature) while keeping other parameters unchanged.

**Fig 6:**
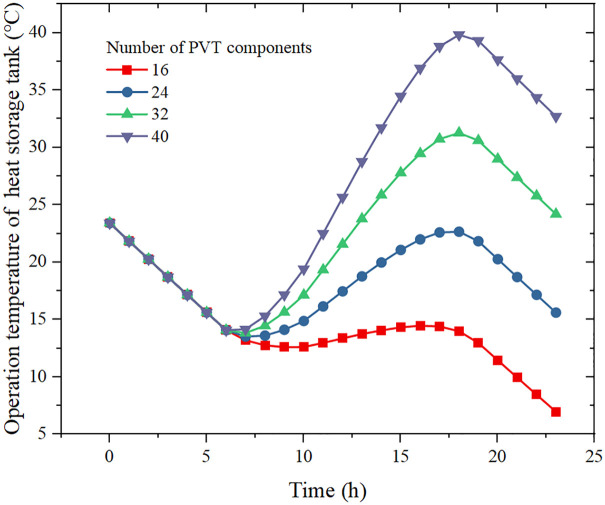
Effect of the number of PVT collector units on the operating temperature of the heat storage tank under specified operating conditions.

Fig 6 presents the variation of storage tank temperature over the typical day for different numbers of PVT modules. Since the heat pump operates continuously, the tank continuously provides heat to the CO_2_ cycle. During periods without solar irradiance (night and early morning), the storage temperature decreases almost linearly due to heat extraction and heat loss. Between approximately 07:00 and 19:00, the PVT field supplies heat to the tank, and the storage temperature increases.

As the number of PVT modules increases, both the rate and the magnitude of temperature rise in the tank become larger. To ensure stable operation over 24 h, the storage temperature at 24:00 should not fall below its value at 00:00. Under this criterion, at least 32 PVT modules are required. For this configuration, the storage temperature varies between 13.8 °C and 31.3 °C during the day.

**Fig 7:**
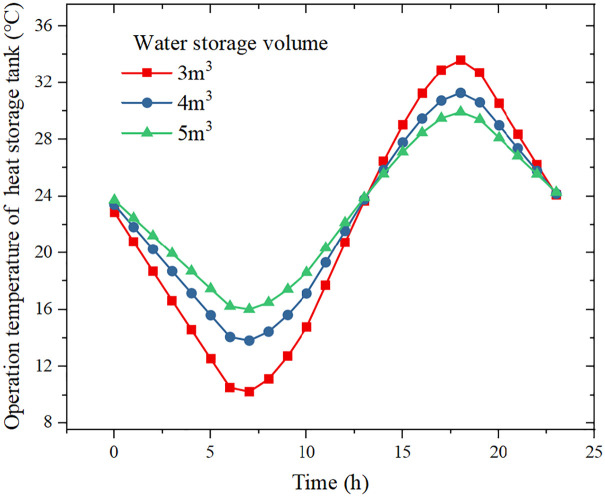
Effect of water storage volume on operation temperature of heat storage tank.

Fig 7 shows the influence of storage tank volume on operation temperature of heat storage tank. The storage volume mainly affects the minimum and maximum operating temperatures. When the storage volume is 5 m³, the minimum and maximum temperatures are 16.0 °C and 29.9 °C, respectively. When the volume is reduced to 3 m³, the minimum and maximum temperatures become 10.2 °C and 33.6 °C, respectively.

A larger storage volume reduces temperature fluctuations and thus enhances system stability, but it also increases investment cost and may lead to lower average evaporation temperature if the tank is oversized. Conversely, a smaller tank results in larger temperature swings, which may cause the evaporation temperature to drop excessively during low-irradiance periods, thereby degrading the COP. Therefore, the storage volume needs to be optimized by considering both performance and cost.

**Fig 8:**
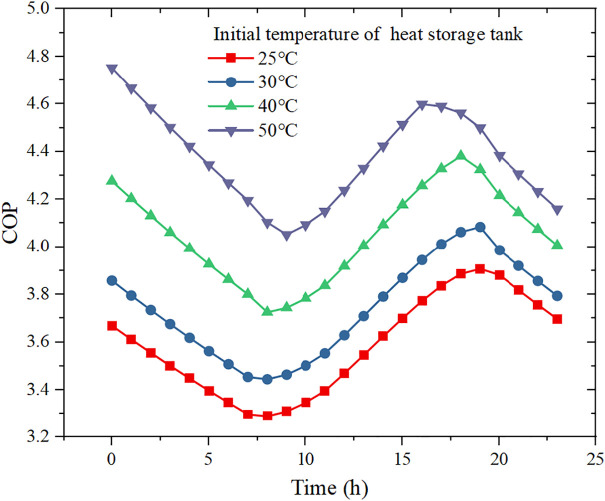
Effect of initial temperature of heat storage tank on COP.

Fig 8 shows that, for a given 10 kW heating load, the COP is strongly governed by the initial storage temperature and follows a characteristic decrease–increase–decrease pattern over the typical day. At the beginning of the simulation, the storage temperature is relatively high and the evaporation temperature of the CO_2_ cycle is favorable, so the COP starts from a higher level. As heat is continuously extracted during the night and early morning while no solar input is available, the storage temperature drops and the evaporation temperature decreases, which increases the compression ratio and causes the COP to decline. After about 8–10 h, solar irradiance becomes sufficiently strong and the PVT field starts to recharge the tank; the storage temperature rises again, alleviating the compression ratio and leading to a gradual recovery of COP. In the late afternoon and evening, the solar input weakens and the tank cools down once more, so the COP decreases again. Increasing the initial storage temperature from 25 °C to 50 °C shifts the entire COP curve upward, and the daily average COP increases from 3.59 to 4.37, highlighting the strong sensitivity of the high-temperature CO_2_ heat pump to the heat-source temperature.

**Fig 9:**
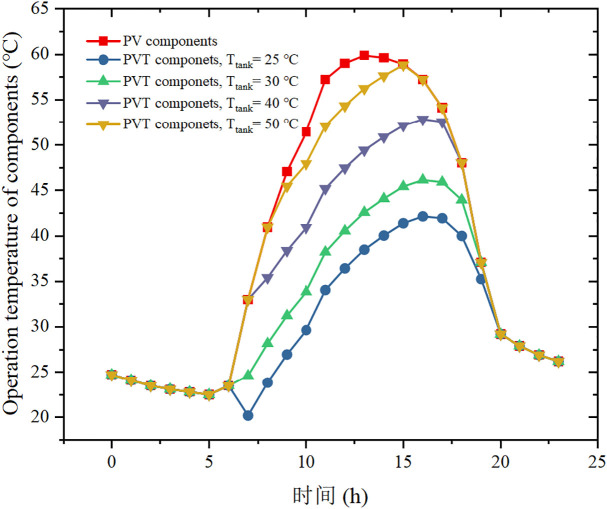
Effect of PV component and PVT component (with different initial temperature of heat storage tank) on operation temperature of components.

Fig 9 compares the operating temperature of a standalone PV module with that of the PVT modules for different initial storage temperatures. The standalone PV module reaches a maximum temperature of 59.9 °C around noon, which significantly penalizes electrical efficiency. In contrast, the PVT configuration keeps the cell temperature noticeably lower due to active water cooling: for an initial storage temperature of 25 °C, the peak PVT module temperature is only 41.9 °C, whereas it increases to 58.8 °C when the initial storage temperature is 50 °C. This behaviour reflects the fact that a colder storage tank provides a larger temperature driving force for heat removal from the PV back surface, thereby enhancing the cooling capacity of the water loop and improving the PVT electrical efficiency. Conversely, when the tank is initially hotter, the reduced temperature difference weakens the cooling effect and leads to higher PV operating temperatures.

Taken together, Fig 8 and Fig 9 reveal an inherent trade-off between thermal and electrical performance. A higher initial storage temperature benefits the heat pump by raising the evaporation temperature and COP, but it simultaneously reduces the temperature gradient for PVT cooling and thus limits the potential electrical efficiency gain. A lower initial storage temperature has the opposite effect, favoring PVT electrical performance but slightly penalizing COP. Therefore, an appropriate combination of storage volume and initial storage temperature is required to balance these competing effects. Rather than maximizing COP or electrical efficiency in isolation, the system should be designed by considering the total energy performance and net daily electricity use, so that overall efficiency is maximized and near net-zero electricity operation can be achieved.

**Fig 10:**
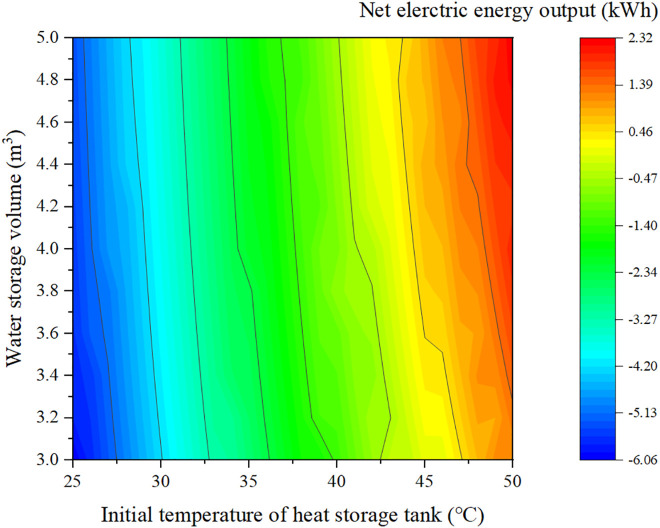
Effect of key heat storage parameters on the net electrical energy output of the system under identical operating conditions.

Fig 10 illustrates the net daily electrical output of the system for different combinations of storage volume and initial storage temperature. For a fixed number of PVT modules, the net electrical output increases with storage volume and significantly increases with initial storage temperature. Within the studied parameter range, the net daily electrical output varies from –6.06 kWh to 2.32 kWh. The maximum value is obtained when the storage volume is 5 m³ and the initial temperature is 50 °C, where the system exports 2.32 kWh of electricity over the typical day.

However, high initial storage temperatures usually require additional energy input at the beginning of operation or on previous days, which may not always be practical. To balance performance and feasibility, a design point with storage volume 4.4 m³ and initial temperature 42 °C is selected, where the net daily electrical output is approximately zero. In other words, the electricity generated by the PVT modules is just enough to cover the compressor consumption, achieving self-sufficient operation.

**Fig 11:**
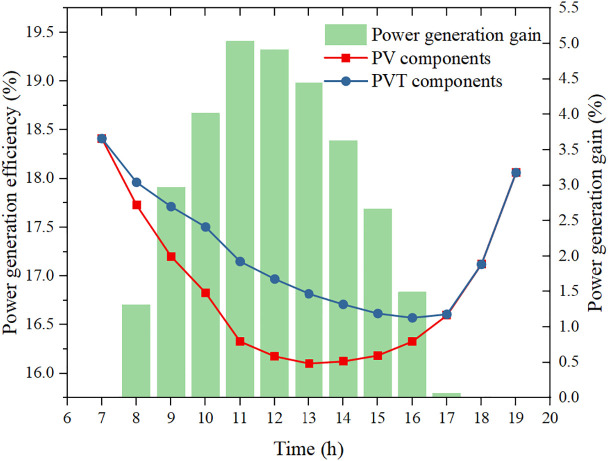
PVT and PV power generation performance during a typical day.

Fig 11 compares the electrical efficiencies of the PVT and standalone PV modules and the corresponding power generation gain under the self-sufficient design condition. Both efficiencies exhibit a typical “U-shaped” daily profile: they are highest in the cool early morning, decrease as rising irradiance drives up cell temperature, and recover in the late afternoon. The minimum efficiency of the standalone PV module drops to 16.1%, while that of the PVT module remains slightly higher at 16.6%, reflecting the cooling effect of the water loop. The resulting instantaneous power generation gain of the PVT configuration reaches a maximum of 5.03% around midday and gradually declines as the storage tank warms up and the cooling potential weakens; when the cooling loop is stopped between 18:00 and 20:00, the gain falls to zero and the PVT behaves like a conventional PV module. These results confirm that, for the net-zero electricity design point, the PVT configuration consistently mitigates the efficiency loss caused by high cell temperatures and yields a positive power generation gain during most of the daytime, even though the absolute efficiency difference at each hour appears modest. When integrated over the full day, this incremental gain in electrical output is sufficient to offset the compressor electricity consumption, enabling self-sufficient operation of the high-temperature CO_2_ heat pump system without increasing the installed PV area.

**Fig 12:**
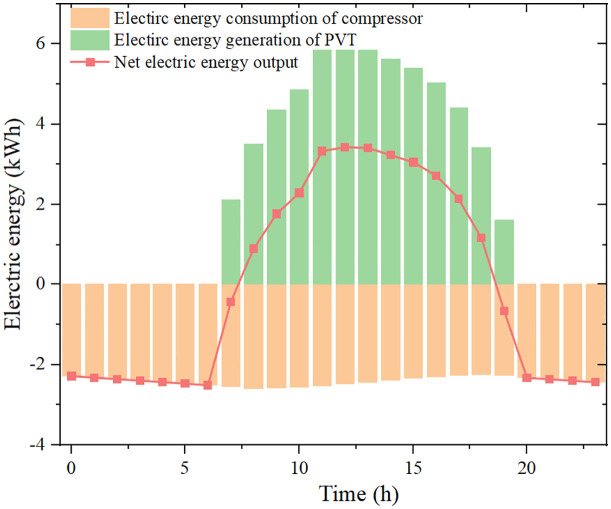
The performances of the proposed system during a typical day.

Fig 12 presents the hourly compressor electricity consumption, PVT electricity generation and net electrical output. The compressor electrical power exhibits a non-monotonic trend of increase–decrease–increase, and ranges from approximately 2.25 kW to 2.60 kW. As the storage temperature rises, the compressor power decreases, with a maximum reduction of about 13.5%. The maximum hourly PVT electrical output reaches 5.91 kWh between 12:00 and 13:00, during which the net electrical output also peaks at 3.43 kWh.

These results demonstrate that an appropriate design of storage volume and initial temperature not only stabilizes high-temperature heat supply but also enables net-zero or even positive electricity generation, enhancing the overall energy and exergy utilization of the system.

**Fig 13:**
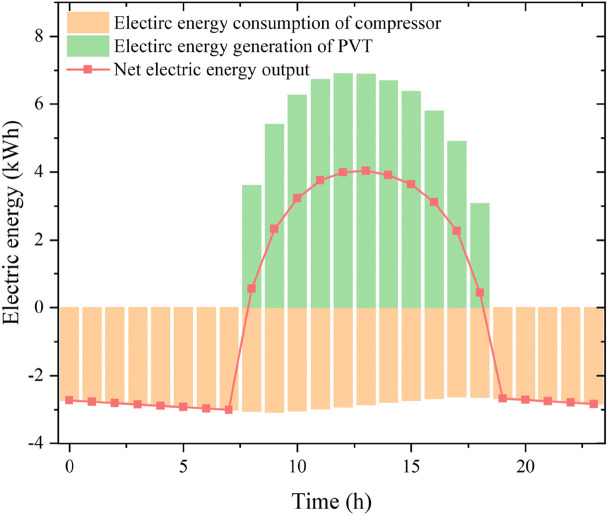
The performances of the proposed system during a spring day.

**Fig 14:**
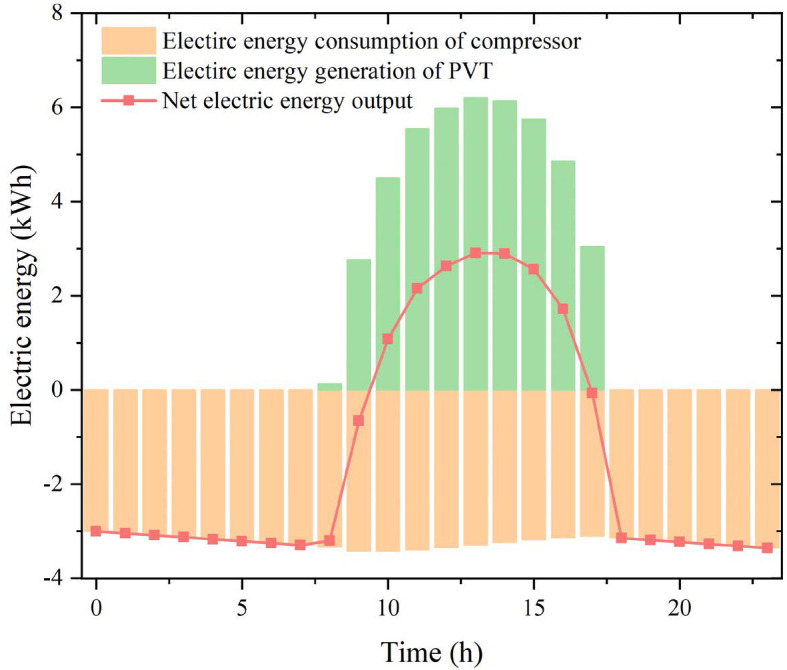
The performances of the proposed system during a winter day.

In addition to the typical day (summer) discussed above, spring and winter cases are further examined, both under sufficiently sunny conditions. The average ambient temperature is 13 °C in spring and 2 °C in winter. All other parameters are kept unchanged; however, to maintain the cooling effect of the PVT field, the initial storage temperature is adjusted to 25 °C for spring and 10 °C for winter. As shown in Figs 13 and 14, the duration of effective solar irradiation directly affects the electricity generation of the PVT modules. Under spring conditions, the longer effective sunshine period enables higher daytime PVT electricity generation and supports a relatively larger net electrical output. In contrast, although winter solar radiation is also sufficient, the shorter effective sunshine duration weakens the cumulative PVT power generation. Meanwhile, the initial storage temperature strongly affects the heat pump COP by determining the heat-source temperature level. As a result, the net electrical energy output deteriorates from-5.40 kWh in spring to −32.69 kWh in winter. These results indicate that, even under sunny conditions, seasonal differences in irradiation duration and storage temperature have a pronounced influence on the electrical self-sufficiency of the proposed system.

## 5. Ethical sustainability considerations

This study presents a technical analysis of the performance of a solar photovoltaic-thermal assisted high-temperature CO_2_ heat pump system with thermal energy storage. While it does not involve ethical considerations concerning human or animal subjects, the technology itself—arising from the interaction between humans and the natural environment—constitutes a deliberate intervention in natural systems. Its development and deployment are guided by the principles of sustainability ethics, representing a tangible application of this ethical framework in engineering practice.

Specifically, the core principle of sustainable development is commonly defined as “development that meets the needs of the present without compromising the ability of future generations to meet their own needs.” [22]. This principle underscores that current development should not deplete or degrade the resources, ecosystems, and environmental conditions essential for the well-being of future generations. Instead, it emphasizes the protection of biodiversity, the long-term availability of resources, and the maintenance of habitable ecosystems. Sustainability ethics extends this concept by framing sustainable development within an explicit ethical dimension. It emphasizes the responsibility to consider long-term environmental and social impacts when choosing technological pathways, formulating policies, and making decisions, thereby promoting a balanced integration of economic benefits, ecological stability, and social welfare [23].

Evaluating the solar PVT-assisted high-temperature CO_2_ heat pump system with thermal energy storage through the lens of sustainability ethics reveals that this technology is designed to harness clean and abundant solar energy to meet human energy needs. Unlike conventional energy extraction methods, such as coal mining or oil drilling, this approach inherently reflects an ethical commitment to sustainable energy use from the outset. It not only satisfies current energy demands but also prioritizes the preservation of resources for future generations, ensuring equitable access to development opportunities. In this way, the system embodies the principle of intergenerational equity in energy utilization. Accordingly, the proposed system represents a practical pathway toward intergenerational energy equity, as it can continuously deliver 10 kW of heat above 100 °C over 24 h and approach net-zero daily electricity use under optimized design conditions.

## 6. Conclusions

A solar PVT-assisted high-temperature CO_2_ heat pump system with hot-water thermal storage has been proposed and analysed for industrial-grade heat supply. A dynamic model coupling the PVT modules, storage tank and transcritical CO_2_ heat pump was developed and applied to a 10 kW continuous heating case under typical-day conditions of Xi’an, China. The main conclusions are summarised as follows:

(1) To ensure stable 24-h operation with a continuous 10 kW heat load, at least 32 PVT modules are required. The storage tank temperature and heat pump COP both exhibit a decrease–increase–decrease pattern over the day. When the initial storage temperature increases from 25 °C to 50 °C, the average COP improves from 3.59 to 4.37.
(2) For a fixed number of PVT modules, the net daily electrical output increases with storage volume and increases markedly with initial storage temperature. Within the studied range, the net electrical output varies from –6.06 kWh to 2.32 kWh, with the maximum obtained at a storage volume of 5 m³ and initial temperature of 50 °C.
(3) A design point with storage volume 4.4 m³ and initial temperature 42 °C achieves net-zero daily electricity consumption: the PVT electricity production fully matches the compressor demand. Under this condition, the maximum instantaneous PVT electrical efficiency gain compared with a pure PV array is 5.03%. The maximum hourly PVT electrical output is 5.91 kWh between 12:00 and 13:00, during which the net electrical output reaches 3.43 kWh.

Overall, the proposed solar PVT-assisted high-temperature CO_2_ heat pump system with thermal storage demonstrates the potential to provide clean and stable industrial heat supply while improving solar energy utilization. Future work will further consider storage stratification, compressor operating constraints, supply-temperature robustness, detailed economic optimization, and experimental validation. In addition, intelligent operation strategies, including emerging quantum-inspired optimization methods [24], may also be explored.

### Highlights

Solar PVT-assisted transcritical CO_2_ heat pump for high-temperature heating.

At least 32 PVT modules are needed to supply 10 kW continuous heat above 100 °C.

Larger storage volume and higher initial storage temperature enhance the net daily electrical output.

A 4.4 m³ tank at 42 °C achieves net-zero daily electricity with a 5.03% PVT efficiency gain.
